# Hippocampal Astrocytes in Migrating and Wintering Semipalmated Sandpiper *Calidris pusilla*

**DOI:** 10.3389/fnana.2017.00126

**Published:** 2018-01-04

**Authors:** Dario Carvalho-Paulo, Nara G. de Morais Magalhães, Diego de Almeida Miranda, Daniel G. Diniz, Ediely P. Henrique, Isis A. M. Moraes, Patrick D. C. Pereira, Mauro A. D. de Melo, Camila M. de Lima, Marcus A. de Oliveira, Cristovam Guerreiro-Diniz, David F. Sherry, Cristovam W. P. Diniz

**Affiliations:** ^1^Laboratório de Investigações em Neurodegeneração e Infecção no Hospital Universitário João de Barros Barreto, Instituto de Ciências Biológicas, Universidade Federal do Pará, Belém, Brazil; ^2^Laboratório de Biologia Molecular e Neuroecologia, Instituto Federal de Educação Ciência e Tecnologia do Pará, Bragança, Brazil; ^3^Department of Psychology, University of Western Ontario, London, ON, Canada; ^4^Advanced Facility for Avian Research, University of Western Ontario, London, ON, Canada

**Keywords:** 3-D glial cell, Nearctic bird, non-stop flight, Bay of Fundy, Canela, stereology

## Abstract

Seasonal migratory birds return to the same breeding and wintering grounds year after year, and migratory long-distance shorebirds are good examples of this. These tasks require learning and long-term spatial memory abilities that are integrated into a navigational system for repeatedly locating breeding, wintering, and stopover sites. Previous investigations focused on the neurobiological basis of hippocampal plasticity and numerical estimates of hippocampal neurogenesis in birds but only a few studies investigated potential contributions of glial cells to hippocampal-dependent tasks related to migration. Here we hypothesized that the astrocytes of migrating and wintering birds may exhibit significant morphological and numerical differences connected to the long-distance flight. We used as a model the semipalmated sandpiper *Calidris pusilla*, that migrates from northern Canada and Alaska to South America. Before the transatlantic non-stop long-distance component of their flight, the birds make a stopover at the Bay of Fundy in Canada. To test our hypothesis, we estimated total numbers and compared the three-dimensional (3-D) morphological features of adult *C. pusilla* astrocytes captured in the Bay of Fundy (*n* = 249 cells) with those from birds captured in the coastal region of Bragança, Brazil, during the wintering period (*n* = 250 cells). Optical fractionator was used to estimate the number of astrocytes and for 3-D reconstructions we used hierarchical cluster analysis. Both morphological phenotypes showed reduced morphological complexity after the long-distance non-stop flight, but the reduction in complexity was much greater in Type I than in Type II astrocytes. Coherently, we also found a significant reduction in the total number of astrocytes after the transatlantic flight. Taken together these findings suggest that the long-distance non-stop flight altered significantly the astrocytes population and that morphologically distinct astrocytes may play different physiological roles during migration.

## Introduction

Many migratory birds return to their breeding territory year after year, which requires a long-lasting memory (Sherry, 2006; Mettke-Hofmann, 2017). The hippocampus seems to be essential in birds for recalling landmarks and migratory routes in long-distance navigation (Mettke-Hofmann and Gwinner, 2003; Mouritsen et al., 2016). The involvement of the hippocampus in these tasks is reflected in the neuroanatomical differences between the hippocampal formation in migratory versus non-migratory bird species (Krebs et al., 1989; Sherry and Vaccarino, 1989; Jacobs et al., 1990; Healy and Krebs, 1996; Garamszegi and Eens, 2004; Lucas et al., 2004; Roth and Pravosudov, 2009; LaDage et al., 2010, 2011; Diniz C.G. et al., 2016). In addition, de Morais Magalhães et al. (2017) demonstrated that the long-distance migratory species *Calidris pusilla* has a significant difference in the number of newborn neurons in migrating and wintering individuals. However, most studies of the role of the hippocampal plastic response in memory formation have focused on the number of neurons and on changes in hippocampal volume, with few reports investigating the role of glial cells and the hippocampus in migration (Healy et al., 1996; Roth et al., 2013). A recent study investigated two sandpiper species with contrasting demands on visuospatial learning during their migrations. One species, *Actitis macularia*, relies more on remembering visual cues during overland migration and has a larger hippocampus and more microglial cells than the other species, *C. pusilla*, which migrates via a long-distance non-stop flight over the Atlantic Ocean (Diniz C.G. et al., 2016). In addition to these indirect findings, Gibbs et al. (2011) demonstrated that glial cells may more directly contribute to long-term memory formation. Indeed, they demonstrated that memory consolidation in chicks is modulated by endogenous hippocampal adenosine triphosphate (ATP) from astrocytes, revealing that astrocytes are important players in memory formation and recollection in birds. In astrocytes, GFAP, together with lesser amounts of vimentin (Eliasson et al., 1999), nestin (Thomsen et al., 2013), and synemin (Jing et al., 2007), are the major intermediate filament proteins that constitute the glial filaments. Antibodies specific to GFAP are important tools to facilitate studies of the normal biology of GFAP (Lin et al., 2017).

Metabolically, long-distance migratory flights require lipid reserves (Landys et al., 2005). In the brain, vast demands are placed on astrocyte metabolism. Accordingly, we hypothesized that long flights may affect the morphology and physiology of astrocytes and that this may be detectable by morphometry. To our knowledge, there are no detailed three-dimensional (3-D) morphological or stereological studies of astrocytes in the hippocampal formation of migratory birds, and no studies have investigated the impact of uninterrupted long-distance migratory flights on astrocytes in the hippocampal formation.

In the present work, we investigated changes in astrocytes in the *C. pusilla* hippocampal formation. Specifically, we compared the morphology and number of these cells in the hippocampal formation of birds captured before and after the transatlantic migratory flight to the northeast coast of South America. We predicted that the astrocytes in the hippocampi of the sandpipers would show clear morphological and numerical differences that might be related to the intense metabolic demands imposed by the non-stop long-distance flight.

## Materials and Methods

Five migrating *C. pusilla* were collected in August 2012 at a stopover site in the Bay of Fundy, Canada (45°50′19.3″ N and 64°31′5.39″ W), and another five were captured in the wintering period, between September and March, on Isla Canela, in the tropical coastal zone of northern Brazil (00°47′09.07″ S and 46°43′11.29″ W). Birds were captured under license N° 44551-2 from the Chico Mendes Institute for Biodiversity Conservation (ICMBio) and Scientific Capture permit ST2783 from the Canadian Wildlife Service. All procedures were carried out in accordance with the Association for the Study of Animal Behavior/Animal Behavior Society Guidelines for the Use of Animals in Research and with approval of the Animal Users Subcommittee of the University of Western Ontario. All efforts were made to minimize the number of animals used and the stress and discomfort to animals.

Semipalmated sandpipers reach the coastal zone of northern Brazil in August and September and begin their migration to the arctic between May and July.

### Perfusion and Histology

Under deep isoflurane anesthesia, the birds were perfused transcardially with 0.1 M phosphate-buffered saline (PBS) followed by aldehyde fixative (4% paraformaldehyde in 0.1 M phosphate buffer, pH 7.2–7.4). The brains were dissected, post-fixed in 4% paraformaldehyde, stored in 0.05 M PBS and sliced using a Vibratome (Leica VT1000S) in the coronal plane into 60-μm thick sections to obtain six anatomical serial sections. The free-floating sections were immunolabeled with anti-GFAP antibody (SC-6170, Santa Cruz Biotechnology) and mounted on glass slides coated with an aqueous solution of gelatin (10%) and chromium potassium sulfate (0.5%). The sections were air-dried at room temperature, dehydrated and cleared using an alcohol and xylene series.

### Immunohistochemistry

Free-floating sections were subjected to antigenic retrieval by incubation in 0.2 M boric acid (pH 9) at 70°C for 60 min, washed in PBS-Triton (PBST; 0.1% Triton) and washed 3 × 2 min in PBS. The sections were then immersed for 12 h in PBST plus 5% normal horse serum and incubated for 12 h at 4°C with the anti-GFAP antibody (SC-6170, Santa Cruz Biotechnology) diluted 1:500 in PBST (0.3% Triton) with gentle and continuous agitation. After washing in PBST (0.1% Triton), the sections were incubated overnight with horse anti-goat secondary antibody (Vector Laboratories, Inc.) diluted 1:400 in PBST (0.3% Triton); incubated in 0.3% hydrogen peroxide for 15 min; washed 3 × 2 min in PBST; and then incubated for 60 min in avidin–biotin–peroxidase complex solution [Vector Laboratories, Burlingame, CA, United States; 37.5 μl A + 37.5 μl B in 13.12 ml PBST (0.3% Triton)]. After a 2-min wash in PBS, the glucose-oxidase-DAB-nickel method (Shu et al., 1988) was used to visualize GFAP-immunolabeled astrocytes. The reaction was stopped after fine astrocytic branches were detected under the microscope. Sections were rinsed 4 × 5 min in 0.1 M PBS, mounted on gelatinized slides, dehydrated in an alcohol and xylene series, and placed on a coverslip with Entellan (Merck). Five animals from each group with complete GFAP immunohistochemistry slide series that contained clear morphological details of astrocytes were used for the 3-D reconstruction and morphometric analyses. We confirmed the specificity of the immunohistochemical pattern using a negative control reaction that omitted the primary antibody (Saper and Sawchenko, 2003). This negative control showed no astrocyte immunolabeling.

### Three-Dimensional Astrocyte Reconstruction and Quantitative Morphology

Brain sections were analyzed with a NIKON Eclipse 80i microscope (Nikon, Japan) equipped with a motorized stage (MAC6000, Ludl Electronic Products, Hawthorne, NY, United States). The astrocytes were imaged with a high-resolution, 100× oil immersion plan fluoride objective (Nikon, NA 1.3, DF = 0.19 μm). Images were acquired and analyzed with Neurolucida Neuron Tracing Software (Neurolucida 11.03; MBF Bioscience, Williston, VT, United States). Although shrinkage in the *z*-axis is not a linear event, we corrected the shrinkage in the *z*-axis based on previous evidence of 75% shrinkage (Carlo and Stevens, 2011). Without correction, this shrinkage would significantly distort the length measurements along this axis. Only cells with processes that were unequivocally complete were included in the 3-D analysis; cells were discarded when the branches appeared to be artificially cut or not fully immunolabeled. The terminal branches were typically thin.

### Morphometric Analysis and Statistics

**FIGURE 1:**
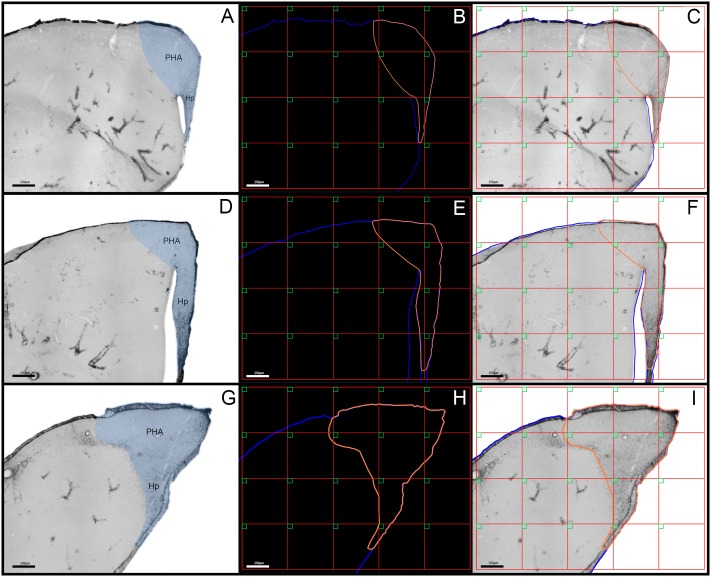
**(A,D,G)** Low-power photomicrographs of the *C. pusilla* hippocampal formation from rostral **(A)**, medial **(D)**, and caudal **(G)** sections that were immunolabeled with anti-GFAP antibody to define the limits of the area of interest and the sampling strategy **(C,F,I)**. The hippocampal formation comprises the hippocampus proper (Hp) and the parahippocampal area (PHA). The hippocampal formation is shown in blue. Intense GFAP immunostaining clearly shows the V region of the hippocampus proper. **(B,E,H)** The red grid establishes the intervals between the square green boxes and illustrates the systematic random sampling approach. **(C,F,I)** The number of boxes in each section was proportional to the area covered by the hippocampal formation. A single astrocyte located inside every box was selected for 3-D reconstruction. Scale bars: 250 μm.

The morphometric analysis was performed on 10 birds, including all five from each group. We performed digital 3-D reconstruction on a total of 499 astrocytes that were selected using a systematic random unbiased sampling approach (Glaser and Wilson, 1998; Glaser and Glaser, 2000) (**Figure 1**). We also estimate the number of total astrocytes in the hippocampal formation in migrating and wintering birds using optical fractionator (West et al., 1991).

Based on previous descriptions (Atoji and Wild, 2006; Atoji et al., 2016), we defined the sandpiper hippocampal formation as comprising the hippocampus proper and the parahippocampal area. The lateral and ventral limits of the hippocampus were defined by the lateral ventricle, the dorsal and caudal limits corresponded to the cerebral surface, the medial limit was defined by the interhemispheric fissure and the inferior limit was defined by a marked change in cell density in the dorsal-most hippocampal “V” region near the septal area (**Figure 1**). The parahippocampal area was located dorsal and lateral to the hippocampus, as defined medially by the paraventricular sulcus (Atoji and Wild, 2006; Atoji et al., 2016). To generate unbiased and statistically valid results, we used systematic random sampling to ensure that all regions of the hippocampal formation would contribute to the sample with the same probability. Thus, systematic random samples were taken from a series of sections containing dorsal and ventral portions of the hippocampal formation. Each box in the outlined hippocampal formation (**Figure 1**) indicates a site from which we selected a single astrocyte for 3-D reconstruction. We used multivariate statistical analyses to compare morphological features of astrocytes in migrating and wintering birds.

**FIGURE 2:**
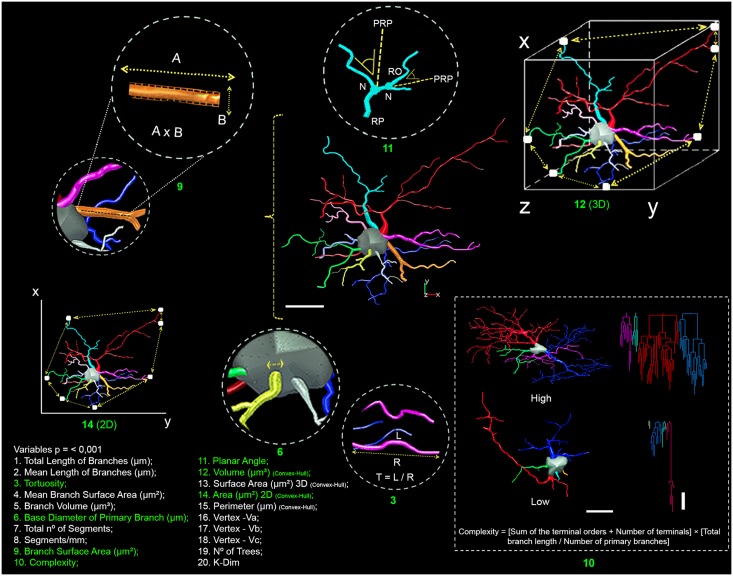
Schematic representation of the morphometric features obtained from the three-dimensional reconstructions. Comparisons of 20 morphometric variables (1–20) revealed significant differences between astrocyte types (Type I versus Type II) and between the experimental groups (migrating versus wintering birds). Scale bars = 10 μm. Green numbers have drawings for better definitions of the variables.

We first investigated shared morphological features in the astrocytes in the two groups. We selected all morphometric quantitative variables with multimodality indices (MMIs) higher than 0.55 for an initial cluster analysis (Ward’s hierarchical clustering method) that included all animals from each group. We estimate the MMI based on the skewness and kurtosis of our sample for each morphometric variable as previously defined elsewhere: MMI = (M3^2^ + 1)/[M4 + 3 (*n* - 1)^2^/(*n* - 2) (*n* - 3)], where M3 is skewness and M4 is kurtosis and *n* is the sample size (Kolb et al., 1994; Schweitzer and Renehan, 1997). Kurtosis and skewness describe the shape of the data distribution and allow us to distinguish between unimodal and multimodal curves. Multimodal data sets are essential for separating a population of cells into cell types (Kolb et al., 1994; Schweitzer and Renehan, 1997). The multimodal index of each variable was estimated based on the measurements of 20 morphometric features of astrocyte branches (**Figure 2**).

We found that a few morphological features of the astrocytes showed an MMI greater than 0.55, indicating that the distribution was at least bimodal and might be multimodal. These features were selected for cluster analysis as described previously (Kolb et al., 1994; Schweitzer and Renehan, 1997). We used Ward’s method with standardized variables and a tree diagram (dendrogram) to illustrate the classification generated by cluster analysis.

Astrocyte classification as suggested by cluster analysis was assessed using a forward stepwise discriminant function analysis performed with Statistica 12.0 (Statsoft, Tulsa, OK, United States). Discriminant function analysis was used to determine which variables discriminate between two or more naturally occurring groups. The purpose of this procedure is to determine whether the groups differ in the mean value of a variable, and then to use that variable to predict group membership. We used Statistica software to perform comparisons between the matrices of total variances and co-variances. These matrices were compared using multivariate *F*-tests to determine whether there were any significance between-group differences (for all variables). In the step-forward discriminant function analysis, the program builds a model of discrimination step-by-step. In this model, at each step, all variables are reviewed and evaluated to determine which variable contributes the most to the discrimination between groups. We used this procedure to identify the morphometric variables that provided the best separation between the astroglial classes that were suggested by the cluster analysis. In addition, we calculated the arithmetic means and standard deviations for the variables chosen as the best predictors for the astroglial groups. We performed a normality test (Shapiro–Wilk) to verify if the data follows a normal distribution. Parametric and non-parametric statistical analyses using *t*-tests (normal distribution) and Mann–Whitney (non-normal distribution) were used to compare groups of astrocytes within each group and to detect possible morphological differences between the average values of morphometric features of our sample of astrocytes from the hippocampal formation of migrating versus wintering groups. All astrocytes from the hippocampal formation were measured multiple times, and dedicated software (Neurolucida Explorer 11.03, MBF Bioscience, Williston, VT, United States) was used to process the data. We applied these procedures to our sample of astrocytes to search for potential astroglial morphological classes within each experimental group.

Mechanical factors associated with vibratome sectioning and the dehydration procedure can affect microscopic 3-D reconstructions and can induce non-uniform shrinkage in the *z*-axis of the sections (Hosseini-Sharifabad and Nyengaard, 2007). Because shrinkage after dehydration in the *x*- and *y*-axis directions is minimal compared with shrinkage in the *z*-axis direction, morphological changes in the *x*- and *y*-axis directions cannot be linearly extrapolated to the *z* dimension. However, the curling of branches is a reliable indication of severe shrinkage in the *z*-axis, as this indicates that the individual processes did not shrink at the same rate as the slice in which they were located. This effect seems greatest at the surface of the slice, decreasing along the *z*-axis. To minimize this differential shrinkage effect, we selected samples from the middle region of the *z*-axis where the impact of these changes is expected to be lower. Recently it was demonstrated that in the *z*-axis (i.e., perpendicular to the cutting surface), the sections shrink by approximately 75% of the cut thickness after dehydration and clearing (Carlo and Stevens, 2011). Based on these findings, all astrocyte reconstructions in our report were corrected for *z*-axis shrinkage of 75% of the original value. Since we expected that the *x*–*y* dimensions would not change substantially after dehydration and clearing, no corrections were applied to these dimensions.

### Stereology

We delineated at all levels in the histological sections the region of hippocampal formation, digitizing directly from sections using low power 4× objective on a NIKON Eclipse 80i microscope (Nikon, Japan), equipped with a motorized stage (MAC6000, Ludl Electronic Products, Hawthorne, NY, United States). This system was coupled to a computer running Stereo Investigator 2017.02 (MBF Bioscience, Williston, VT, United States) used to store and analyzed *x*, *y*, and *z* coordinates of digitized points. In order to detect and count unambiguously the objects of interest in the dissector probe, low power objective was replaced by a plan fluoride objective (Nikon, NA 1.3, DF = 0.19 μm) to count GFAP astrocytes. Thus, all stereological estimations started with the delimitation of the region of interest in coronal sections where the limits of hippocampal formation were unambiguously identified and outlined. At each counting site, the thickness of the section was carefully assessed using the high-power objective and the fine focus of the microscope to define the immediate defocus above (top of section) and below (bottom). Because both the thickness and the distribution of cells in the section were uneven, we estimated the total number of objects of interest based on the number weighted section thickness. We have used selective GFAP marker of astrocytes in anatomical serial of sections to unambiguously distinguish all objects of interest. All sampled objects that came into focus inside the counting frame were counted and added to the total marker sample, provided they are entirely within the counting frame or intersects the acceptance lines without touching the rejection lines (Gundersen and Jensen, 1987). The counting boxes were random and systematically placed within a grid previously defined. **Supplementary Tables S1–S4** show experimental parameters from the optical fractionator of GFAP immunolabeled astrocytes of migrating and wintering *C. pusilla*. These grid sizes were adopted to achieve an acceptable coefficient of error (CE). The calculation of the CE for the total cell counts of each subject in the present study adopted the one-stage systematic sampling procedure (Scheaffer CE) that has been used previously and validated elsewhere (Glaser and Wilson, 1998). The level of acceptable errors of the stereological estimations was defined by the ratio between the intrinsic error introduced by the methodology and the coefficient of the variation (Glaser and Wilson, 1998; Slomianka and West, 2005). The CE expresses the accuracy of the cell number estimates, and a value of CE ≤ 0.05 was deemed appropriate for the present study because variance introduced by the estimation procedure contributes little to the observed group variance (Slomianka and West, 2005). The experimental parameters for each cell marker and regions were established in pilot experiments and uniformly applied to all animals for each marker.

The determination of cell number in the optical fractionator method is based on a random and systematic distribution of counting blocks in a series of section containing the region of interest, all of them with the same probability of been sampled. The optical fractionator determines the number of cells multiplying the number of objects identified inside each counting box by the values of three ratios: (i) the ratio between the number of sections sampled and the total number of sections (section sampling fraction, ssf); (ii) the ratio of the counting box and the area of the grid (area sampling fraction, asf); and (iii) the ratio between the height of the counting frame and the section thickness after histological procedures (thickness sampling fraction, tsf). Thus, the total number of cells for each marker was obtained by the following equation:

N=ΣQ×1/ssf×1/asf×1/tsf

Where, *N* is the total number of cells and ΣQ is the number of counted objects (West et al., 1991).

## Results

### Morphology of *C. pusilla* Hippocampal and Parahippocampal Astrocytes: Qualitative Analysis

**FIGURE 3:**
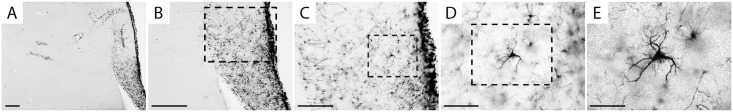
Hippocampal formation brain section photo from a *C. pusilla*, captured on the coast of Bragança, Pará, Brazil, show a stellate astrocyte from the gray matter of the hippocampal V region. Scale bars: **(A)** 250 μm, **(B)** 250 μm, **(C)** 120 μm, **(D)** 60 μm, and **(E)** 25 μm.

**FIGURE 4:**
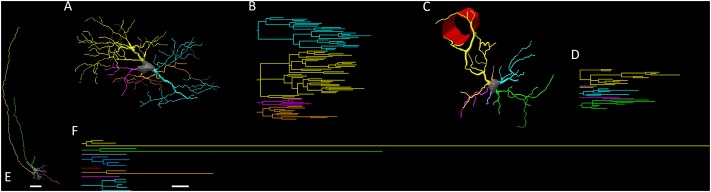
Three-dimensional reconstructions and the respective dendrograms of stellate **(A,B)**, vascular **(C,D)**, and radial **(E,F)** astrocytes. Radial astrocytes were not included in our analysis.

**Figure 3** shows different magnifications of a GFAP-immunolabeled stellate astrocyte from the hippocampus of *C. pusilla* to illustrate the morphological features of the astrocyte. Under a 100× oil immersion objective, all morphological astrocyte details that could aid in 3-D microscopic reconstructions were digitized and stored as *x*, *y*, and *z* coordinates. This procedure identified two other distinct glial cell types, namely radial glia and blood vessel-associated astrocytes, which were GFAP-positive in the *C. pusilla* hippocampal formation (**Figure 4**).

**Figure 4** shows the 3-D reconstructions of astrocytes with three different morphologies associated with distinct physiological roles: radial astrocytes are associated with neurogenesis and neuronal migration (Kempermann et al., 2015); vascular astrocytes are associated with the blood–brain barrier (BBB) inside the neurovascular unit; and stellate astrocytes are more connected to the astrocyte network and less connected to blood vessels (Matyash and Kettenmann, 2010; McConnell et al., 2017).

### Quantitative Analysis of Three-Dimensional Hippocampal and Parahippocampal Reconstructed Astrocytes

**FIGURE 5:**
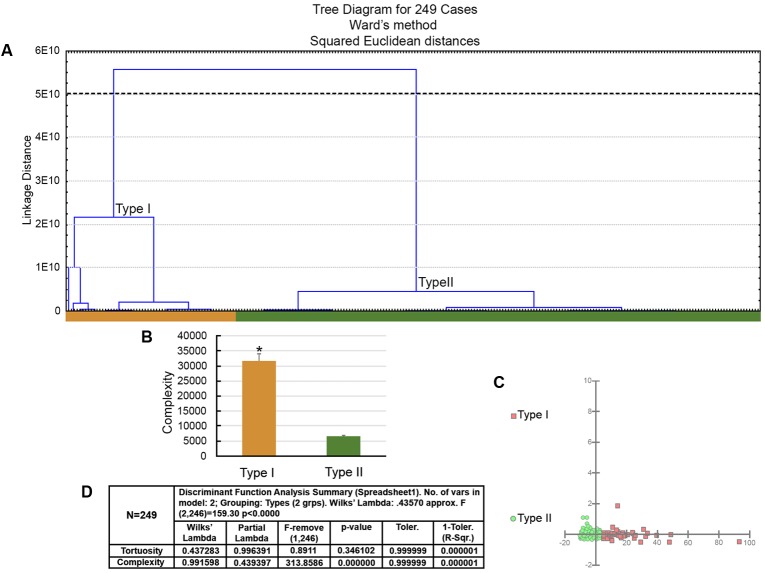
The morphological phenotypes of astrocytes in the hippocampal formation of *C. pusilla* migrating birds. Cluster discriminant analysis (Ward’s method) was performed after three-dimensional reconstruction of astrocytes from five birds. **(A)** Dendrogram groupings of 249 astrocytes identified two main morphological phenotypes, Type I and Type II. **(B)** Graphic representation of complexity mean values and corresponding standard errors illustrates the significant differences between Type I and Type II astrocytes. ^∗^ means there is a significant difference between Type I and Type II astrocytes. **(C)** Graphic representation of discriminant analysis. Note higher dispersion of red filled square corresponding to Type I astrocytes. **(D)** Discriminant statistical analysis results. The variable that contributed the most to cluster formation was complexity (*p* < 0.000). Type I astrocytes (red filled square) showed higher *x*–*y* dispersion than Type II astrocytes (green filled circles). Astrocytes were reconstructed from the rostral to the caudal regions of the hippocampal formation; cluster analysis was based on multimodal or at least bi-modal morphometric features of the astrocytes (MMI > 0.55).

**FIGURE 6:**
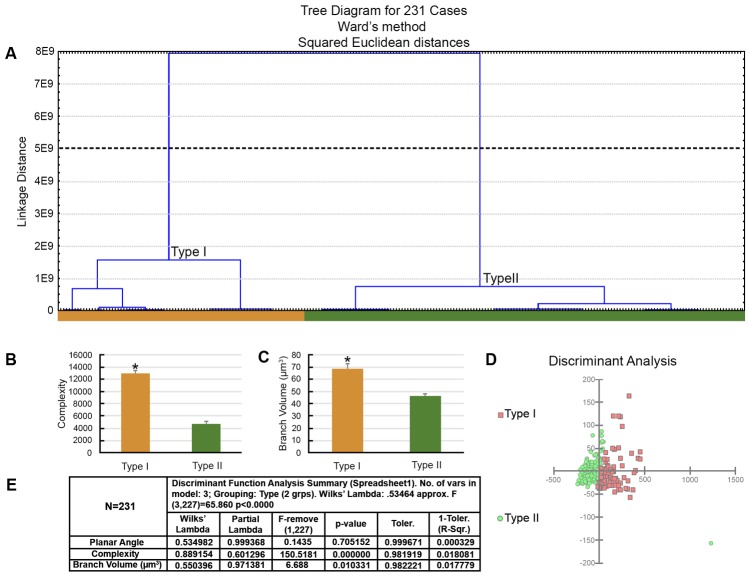
The morphological phenotypes of astrocytes in the hippocampal formation of *C. pusilla* wintering birds. Cluster discriminant analysis (Ward’s method) was performed after three-dimensional reconstructions of astrocytes from five birds. **(A)** Dendrogram groupings of 231 astrocytes identified two main morphological phenotypes, Type I and Type II. **(B,C)** Graphic representation of complexity and branch volume mean values and corresponding standard errors illustrate the significant differences between Type I and Type II astrocytes. ^∗^ means significant statistical diferences. **(D)** Graphic representation of the discriminant analysis. The variable that contributed the most to cluster formation was complexity (*p* < 0.000). Type I astrocytes (red filled square) showed higher *x*–*y* dispersion than Type II astrocytes (green filled circles). Astrocytes were reconstructed from both the rostral and caudal regions of the hippocampal formation; cluster analysis was based on multimodal or at least bi-modal morphometric features of astrocytes (MMI > 0.55). **(E)** Discriminant statistical analysis results.

We performed 3-D reconstructions of cells from the hippocampal and parahippocampal regions of *C. pusilla* but radial astrocytes were not included in our analysis. Based on bimodal or multimodal 3-D morphological features (MMI > 0.55), we searched for morphological families of astrocytes using hierarchical cluster analysis. Independent of the origin of the sample (migrating Bay of Fundy, Canada; wintering Bragança, Brazil), the results showed two families of astrocytes that we designated Type I and Type II (**Figures 5**, **6**) that had remarkable differences in morphological complexity. Astrocytes morphological classification using Type I and Type II designations was based only on their morphological complexities. We designated as Type I astrocytes of the group of cells with higher complexity mean values than that of Type II group (lower complexities). Thus, Type I astrocytes had processes with significantly higher complexity values and more branches than Type II astrocytes. Type I astrocytes also had arbors with more nodes; a higher density of segments/mm; larger branch volumes, branch angles, and tree surface area; and longer total branch length. Type I astrocytes were morphologically more complex than Type II astrocytes in both migrating and wintering birds.

**FIGURE 7:**
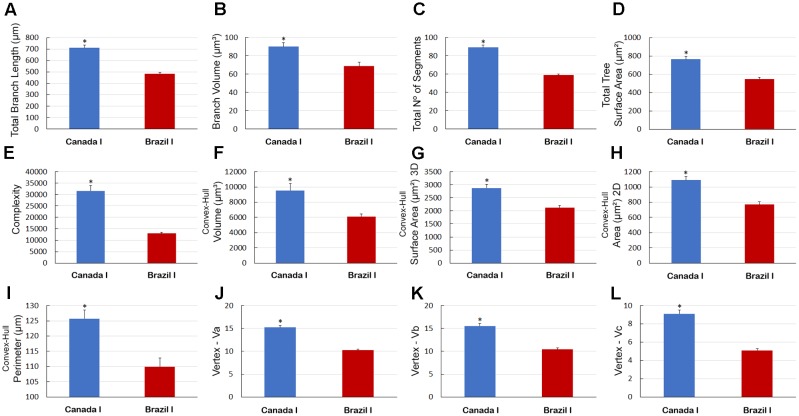
Morphometry of astrocytes that were three-dimensionally reconstructed from the hippocampal formation of five *C. pusilla* individuals captured on the Bay of Fundy, Canada (migrating birds). **(A–L)** Graphic representations show the mean values and standard errors for 12 morphological parameters in Type I astrocytes. Note that after the transatlantic flight, the astrocytes in wintering birds showed shorter total length; lower branch volume; a reduced number of segments and branch surfaces; were less complex; had reduced volume, surface, area, and perimeter of convex-hull and had fewer vertices (Va, Vb, and Vc). Asterisk “^∗^” indicates statistical significant differences.

**FIGURE 8:**
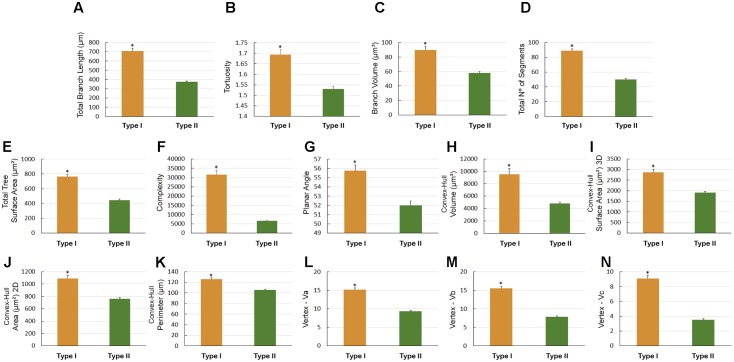
Morphometry of astrocytes that were three-dimensionally reconstructed from the hippocampal formation of 5 *C. pusilla* individuals captured on the Bay of Fundy, Canada (migrating birds). **(A–N)** Graphic representations show the mean values and standard errors for 14 morphological parameters in Type I and II astrocytes. Asterisk “^∗^” indicates statistical significant differences.

There were other significant differences in morphological parameters in type I astrocytes in wintering and migrating birds sampled in Canada and Brazil (**Figure 7**) and between astrocyte types I and II of migrating individuals (**Figure 8**). Indeed, the arbors of migrating animal’s astrocytes had significantly higher values for their total branch length, branch volumes, number of segments, surface areas, complexity, convex-hull volumes, area, surface area, and perimeter and vertexes (**Figure 7**). In addition, total branch length, tortuosity, branch volume, number of segments, surface area, complexity, planar angle, convex-hull volume, surface area, area, and perimeter as well as the vertexes of type I astrocytes have significant higher values (**Figure 8**).

**FIGURE 9:**
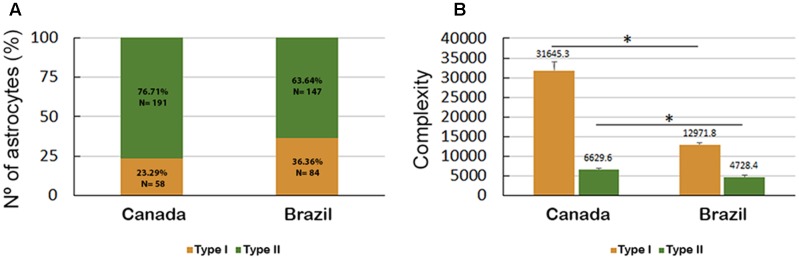
**(A)** Percentage and number of Type I and Type II astrocytes in *C. pusilla* migrating (Canada) and wintering (Brazil) birds and **(B)** astrocyte complexity. The bars in **(B)** show the means and standard error values. Note that Type II astrocytes were less complex than Type I astrocytes, were less affected by the long-distance flight and accounted for a higher proportion of the astrocyte population both in migrating (Canada) and wintering (Brazil) birds. ^∗^ means significant statistical diferences.

Both Type I and Type II astrocytes from the hippocampal formations of migrating birds showed greater morphological complexity than the corresponding Type I and Type II astrocytes from wintering birds (**Figure 9**). Indeed, the Type I astrocytes of migrating birds were, on average, 2.4 times more complex than the Type I astrocytes from wintering birds, and the Type II astrocytes of migrating birds were, on average, 1.4 times more complex than the Type II astrocytes from wintering birds.

Interestingly, the proportion of Type II astrocytes relative to the total number of reconstructed astrocytes was decreased from 76.7% before the transatlantic flight, as seen in migrating birds to 63.6% after the long flight as seen in wintering birds; however, the number of Type I astrocytes increased from 23.3% in migrating birds to 36.4% in wintering birds (**Figure 9**).

**FIGURE 10:**
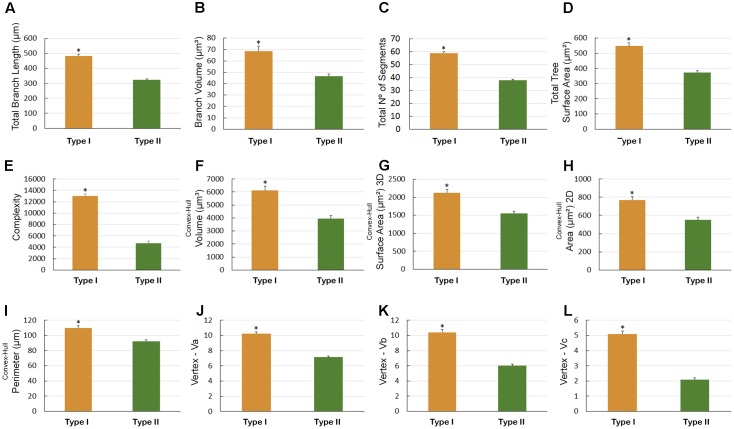
Astrocytes Types I and II morphometry that were three-dimensionally reconstructed from the hippocampal formation of five *C. pusilla* birds captured on Isla Canela, Bragança, Brazil (wintering birds). **(A–L)** Graphic representation of the mean and standard error values of 12 morphological parameters of Type I and II astrocytes. Asterisk “^∗^” indicates statistical significant differences.

Notably, the transatlantic long-distance non-stop flight had a differential effect on the complexity of Type I and Type II astrocytes. Indeed, the complexity of Type I astrocytes seemed to be much more affected by the transatlantic journey than the complexity of Type II astrocytes, and many other morphological features were also more affected in Type I astrocytes than in Type II astrocytes of wintering birds (**Figures 9B**, **10**).

**FIGURE 11:**
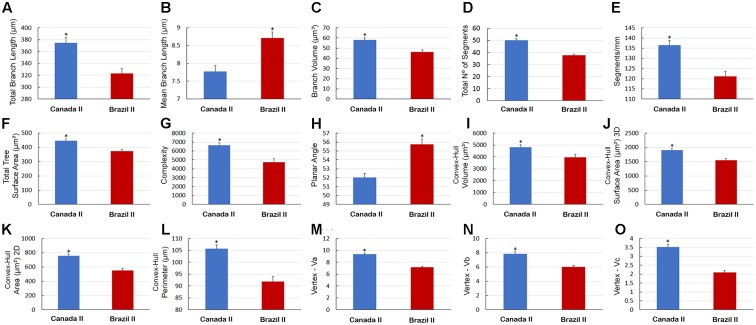
Morphometry of Type II astrocytes that were three-dimensionally reconstructed from the hippocampal formation of five *C. pusilla* birds captured on the Bay of Fundy, Canada and on Isla Canela, Bragança, Brazil. **(A–O)** Graphic representation of the mean and standard error values of 15 morphological parameters of Type II astrocytes. Apart from the mean branch length and planar angle values, which tended to increase after the long flight, the mean values of all other features tended to be reduced in wintering birds. Asterisk “^∗^” indicates statistical significant differences.

Graphical representation of the differences in the morphological features of Type I and Type II astrocytes from wintering birds (**Figure 10**) and in type II astrocytes from migrating and wintering birds (**Figure 11**). Note that after the long-distance flight, both types of astrocytes decreased in size. Indeed, wintering birds showed astrocytes with shorter branches, number of segments, segments/mm, smaller areas and volumes, complexity, convex-hull measurements, and fewer vertices. Exceptions to this rule were that, compared with migrating birds, the wintering birds showed higher mean values for Type II astrocyte branch length and planar angle (**Figure 11B**).

We validated these cluster analyses results using PERMANOVA, a multivariate statistical analysis designed for samples without normal distributions. This test applied to all morphological variables comparing astrocyte types and the migrating windows confirmed significant differences between astrocytes for migrating and wintering birds (*p* = 0.001), as well as between astrocytes types I and II (*p* = 0.001) (please see **Supplementary Data Sheet S2**).

### Morphological Distinction of Hippocampal and Parahippocampal Astrocytes in Migrating and Wintering Birds

**FIGURE 12:**
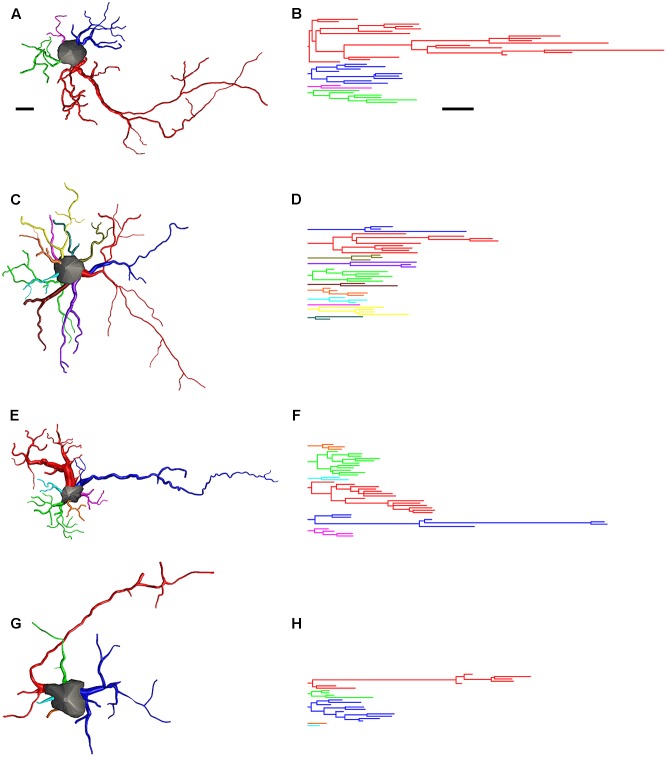
Three-dimensional reconstructions and correspondent dendrograms of Type I **(A,B)** and Type II astrocytes **(C,D)** from migrating birds and Type I **(E,F)** and Type II astrocytes **(G,H)** from wintering birds. Dendrogram were plotted and analyzed with Neurolucida Explorer (MBF Bioscience, Williston, VT United States). Branches of the same parental (primary branch) trunk are shown in one color. As compared to migrating, wintering birds show significant shrinkage of astrocytes branches. Scale bar are the same for **(A–H)** 10 μm.

**Figure 12** shows 3-D reconstructions from microscopic images that illustrate the impact of the transatlantic flight on astrocyte morphology. The 3-D reconstructed cells were selected to show morphometric features that are typical (in terms of mean values) of the Type I and Type II astrocytes we observed in migrating and wintering birds. The 3-D reconstructions and corresponding dendrograms show that in general, before the long-distance non-stop flight, astrocytes are on average much more ramified than astrocytes after migratory flight. **Figure 12** also shows that long-distance flight affected the morphology of Type I astrocytes much more than Type II astrocytes.

To further investigate these changes, we estimated how many of the reconstructed astrocytes from birds captured in Brazil unequivocally exhibited blood vessel connections and identified their morphological class (Type I or Type II). We found that most Type II astrocytes from birds captured after the long flight were connected to blood vessels and that a greater percentage of them had preserved their original (pre-flight) morphology compared to Type I astrocytes.

**FIGURE 13:**
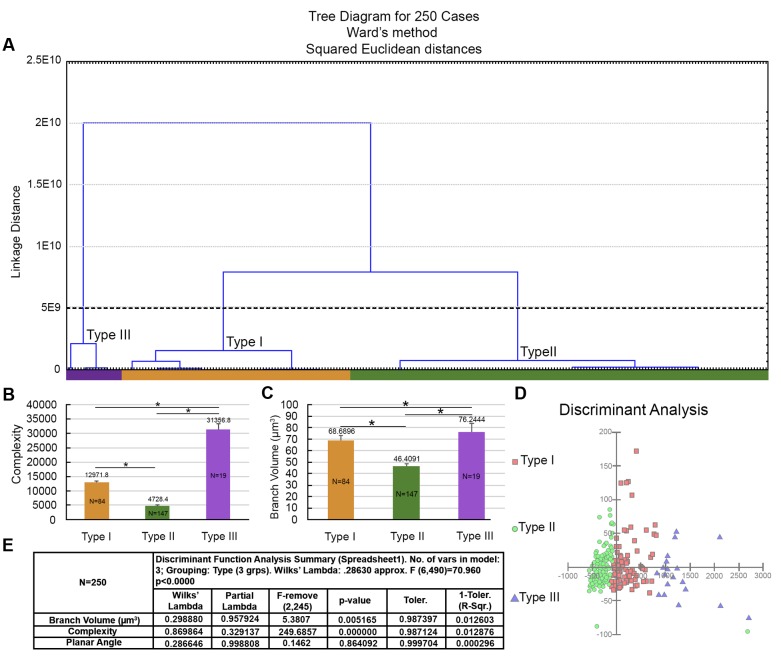
Morphological phenotypes of astrocytes in the hippocampal formation of *C. pusilla* wintering birds. Cluster discriminant analysis (Ward’s method) was applied to three-dimensional reconstructions of astrocytes from five birds. **(A)** Dendrogram groupings of 250 astrocytes show three main morphological phenotypes (Type I, Type II, and Type III). **(B,C)** Graphic representation of the mean complexity and branch volume values and standard errors to illustrate the significant morphological differences between the morphological phenotypes of birds captured before (Canada) and after (Brazil) a long-distance transatlantic flight. ^∗^ means significant statistical diferences. **(D)** Graphic representation of the discriminant analysis. Complexity was the variable that contributed most to cluster formation (*p* < 0.000). Type I astrocytes (red filled square) showed higher *x*–*y* dispersion than Type II astrocytes (green filled circles). The astrocytes were reconstructed from both the rostral and caudal regions of the hippocampal formation; the cluster analysis was based on multimodal or at least bi-modal morphometric features of the astrocytes (MMI > 0.55). Note that the small cluster (19 cells) of Type III astrocytes was not distinguishable when the outliers were removed from the sample.

In addition, to further investigate whether all Type I astrocytes had complexity reduced after the long flight, we applied hierarchical cluster analysis to the entire sample of reconstructed astrocytes from birds captured in Brazil, including outliers that were previously removed. We found a small population of astrocytes (less than 8%, *N* = 19; “Type III” astrocytes) in birds captured in Brazil that, on average, showed morphological complexity that was like that of Type I astrocytes from birds captured before the transatlantic flight (**Figure 13**). See **Table 1** for numerical details.

**Table 1:** The number and percentage of astrocytes interacting with blood vessels in the hippocampal formation of *C. pusilla*.

	Migrating birds		Wintering birds	
		
	*N*	%	*N*	%
Type I	21	20	27	35
Type II	84	80	41	53
Type III	–	–	9	12
Total	105	100	77	100


Similarly, the mean branch volume in Type III astrocytes from the hippocampal formation in wintering birds resembled the mean branch volumes of Type I astrocytes in migrating birds.

### Number of Astrocytes and Volumes of Hippocampal and Parahippocampal Regions and Telencephalon of Migrating and Wintering *C. pusilla*

**Supplementary Tables S1–S4** exhibit all stereological parameters used to count astrocytes with optical fractionator. **Table 2** shows the total number of GFAP positive hippocampal astrocytes of migrating and wintering birds and **Table 3** shows volumes of the hippocampal formation and telencephalon in both hemispheres. We found a significant reduction in the total number of wintering bird’s astrocytes compared to migrating birds in both hemispheres (RHF migrating, *n* = 150,217 ± 30,164 versus RHF wintering *n* = 50,423 ± 14,203; LHF migrating, *n* = 152,237 ± 39,004 versus LHF wintering, *n* = 49,614 ± 17,624). Indeed, migrating birds had 2.76 (LHF) to 2.98 (RHF) times, more astrocytes than wintering birds (two tail *t*-tests, *p* = 0.0007; *t* = 5.36 for LHF and *p* = 0.0002; *t* = 6.69 for RHF), and these changes were not accompanied by changes in hippocampal volume that remained unaltered. As expected, similar differences were found for cell density estimations of hippocampal GFAP immunolabeled astrocytes in both hemispheres, in migrating and wintering bird’s hippocampus.

**Table 2:** Stereological results of GFAP positive astrocytes on the left hippocampal formation (LHF) and right hippocampal formation (RHF) of migrating and wintering *C. pusilla*.

	Capture date	GFAP LHF	SCE LHF	Thickness (μm) LHF	GFAP/mm^3^ LHF	GFAP RHF	SCE RHF	Thickness (μm) RHF	GFAP/mm^3^ RHF
**Migrating**				
*C. pusilla* 10	August 04, 2012	135,733.81	0.03	26.4	22,126.66	134,630.95	0.03	26.7	23,028.01
*C. pusilla* 12	August 04, 2012	101,521.00	0.03	28.1	17,364.70	121,941.35	0.04	29.3	13,680.37
*C. pusilla* 14	August 04, 2012	186,233.47	0.02	26.9	29,326.26	200,939.44	0.02	27,6	36,721.39
*C. pusilla* 16	August 04, 2012	141,162.76	0.04	27.8	29,616.22	147,468.72	0.03	29,4	28,368.10
*C. pusilla* 21	August 06, 2012	196,536.56	0.02	27.4	31,303.61	146,110.63	0.04	26,9	26,913.98
Mean		152,237.52	0.03	27.3	25,947.49	150,218.22	0.037	28.0	25,742.37
SD		39,004.22	0.007	0.68	5,954.55	30,164.63	0.01	1.29	8,391.45
CV^2^		0.065641				0.040322			
CE^2^		0.001239				0.001369			
CVB^2^		0.064402				0.038953			
CVB^2^/CV^2^		0.981124				0.966048			
CVB^2^(%CV^2^)		98.11241				96.60489			
**Wintering**									
*C. pusilla* 82	March 03, 2009	46,428.60	0.03	27.20	14,069.27	56,020.57	0.03	26.80	11,719.78
*C. pusilla* 100	November 10, 2014	55,787.59	0.04	25.40	10,198.83	53,856.90	0.04	26.30	10,880.18
*C. pusilla* 102	November 10, 2014	49,026.91	0.03	26.00	7,708.63	52,460.95	0.06	27.50	10,266.33
*C. pusilla* 105	September 12, 2015	72,817.91	0.03	25.30	14,084.70	63,578.57	0.03	24.70	16,428.57
*C. pusilla* 89	January 14, 2014	24,010.78	0.05	27.10	3,141.95	26,202.79	0.04	25.70	3,738.98
Mean		49,614.36	0.04	26.20	9,840.68	50,423.96	0.04	26.20	10,606.77
SD		17,624.92	0.01	0.91	4,621.68	14,203.57	0.01	1.07	4,541.18
CV^2^		0.126194				0.079345			
CE^2^		0.001600				0.001600			
CVB^2^		0.124594				0.077745			
CVB^2^/CV^2^		0.987321				0.979835			
CVB^2^(%CV^2^)		98.732113				97.983497			


*SCE, Scheaffer coefficient of error; SD, standard deviation.*

**Table 3:** The volume estimates for the left hippocampal formation (LHF), right hippocampal formation (RHF), telencephalon, and the ratio between them for migrating and wintering *C. pusilla*.

	Capture date	Estimated Vol. (mmł) LHF	CE Gundersen *m* = 1 LHF	Estimated Vol. (mmł) LT	CE Gundersen *m* = 1 LT	Vol. LHF/Vol. LT	Estimated (mmł) RHF	CE Gundersen *m* = 1 RHF	Estimated Vol. (mmł) RT	CE Gundersen *m* = 1 RT	Vol. RHF/Vol. RT
**Migrating**				
*C. pusilla* 10	August 04, 2012	6.13	0.016	95.4	0.004	0.064	5.85	0.015	101.32	0.006	0.058
*C. pusilla* 12	August 04, 2012	5.85	0.014	103.45	0.005	0.057	8.91	0.012	102.24	0.005	0.087
*C. pusilla* 14	August 12, 2012	6.35	0.015	94.28	0.002	0.067	5.47	0.016	99.88	0.005	0.055
*C. pusilla* 16	August 07, 2012	4.77	0.019	85.90	0.005	0.055	5.20	0.017	80.44	0.004	0.065
*C. pusilla* 05	August 07, 2012	6.28	0.015	71.37	0.008	0.088	5.43	0.016	79.36	0.006	0.068
Mean		5.88	0.016	90.08	0.005	0.066	6.17	0.015	92.65	0.005	0.067
*SD*		0.65	0.002	12.17	0.002	0.013	1.55	0.002	11.67	0.001	0.013
**Wintering**											
*C. pusilla* 82	March 03, 2009	3.3	0.029	91.7	0.008	0.036	4.78	0.023	76.3	0.008	0.063
*C. pusilla* 100	November 10, 2014	5.47	0.023	77.53	0.017	0.071	4.95	0.019	103.14	0.012	0.048
*C. pusilla* 102	November 10, 2014	6.36	0.018	111.63	0.006	0.057	5.11	0.023	108.08	0.009	0.047
*C. pusilla* 105	September 12, 2015	5.17	0.013	97.42	0.005	0.053	3.87	0.016	88.98	0.006	0.043
*C. pusilla* 89	January 14, 2014	7.64	0.016	131.48	0.004	0.058	7.008	0.018	126.989	0.004	0.055
Mean		5.588	0.020	101.95	0.008	0.055	5.144	0.020	100.698	0.008	0.051
*SD*		1.600	0.006	20.540	0.005	0.012	1.148	0.003	19.263	0.003	0.008


*RT, right telencephalon; LT, left telencephalon; CE, coefficient of error; Vol., volume.*

## Discussion

The avian hippocampus seems to be essential for the integration of multisensory spatial information for navigation, and astrocytes may participate in this task by responding to synaptic activity and to neuronal metabolism. Here we measured the impact of a non-stop flight over the Atlantic Ocean on the morphology *C. pusilla* astrocytes. Specifically, we compared 3-D reconstructions of astrocytes from the hippocampal formations of migrating versus wintering birds. Based on hierarchical cluster analysis of astrocyte morphological features, we categorized the astrocytes into two groups, designated Type I and Type II astrocytes, and analyses showed that complexity was the morphometric feature that best distinguished these two groups. We also found that Type I astrocytes were more affected than Type II astrocytes by the transatlantic flight, suggesting that these populations may have distinct physiological roles that make them more susceptible to the effects of a long flight. Because most Type II astrocytes were physically closer to cerebral blood vessels than Type I astrocytes, we suggest that Type II astrocytes may be more involved in the function of the neurovascular unit and, as such, their bioenergetics and redox activity may protect them so that they can survive and function during the transatlantic non-stop flight.

### The Morphology of Type II Astrocytes Is Less Affected than the Morphology of Type I Astrocytes by the Non-stop Transatlantic Flight

Hierarchical cluster analysis was applied to the morphological features of astrocytes in the hippocampal formation before and after the long-distance flight. Statistical comparative analysis of astrocyte morphologies showed that after transatlantic flight occurred a reduction of the morphological complexities of Type I and Type II astrocytes but to a very different extent. We speculated that the less complex morphological phenotypes observed after the long flight, were due to alterations in the phenotypes of the pre-flight astrocyte families found in individuals captured on Bay of Fundy.

### Possible Implications of the Effects of Non-stop Transatlantic Flight on *C. pusilla* Hippocampal Astrocyte Physiology

No previous reports have described astrocytes changes in shorebirds after long-distance non-stop flights, and never have 3-D reconstructions of astrocytes and a stereological unbiased sampling approach been used to quantify such changes. Here we compared the 3-D morphology of astrocytes in migrating versus wintering birds using hierarchical cluster analysis to classify the cells. We discovered two types of astrocytes with morphological phenotypes that were differentially affected by the long-distance non-stop transatlantic flight. The branches of the two types of astrocytes shrunk to different extents after the long flight, and the two types also showed differences in terms of the percentage of cells that were connected to blood vessels. Because a greater percentage of Type II astrocytes (72.5%) interacted with blood vessels compared to Type I astrocytes (27.5%), both in migrating and in wintering birds, and because this interaction may reflect their relative contribution to the neurovascular unit, we hypothesize that Type II astrocytes may be more involved in the BBB than Type I astrocytes. The BBB includes brain microvascular endothelial cells, astrocytes, neurons, and pericytes, all of which form the neurovascular unit (Weiss et al., 2009; Almutairi et al., 2016; Canfield et al., 2016; McConnell et al., 2017). Because the long flight differentially affected Type I versus Type II astrocytes, we suggest that these cells may have distinct physiological roles. If more Type II astrocytes are indeed, more involved in the neurovascular unit than Type I astrocytes, then the integrity of Type II astrocytes are likely be essential to the integrity of the BBB, and their morphology should be conserved even in adverse conditions. The BBB forms a physical and metabolic barrier between the blood and the brain and helps determine the polarity of astrocyte control of blood flow over arterioles in response to bioenergetic demands (Gordon et al., 2008). To guarantee the integrity of the BBB, we suggest that Type II astrocytes may increase redox activity levels via alternative metabolic pathways to control blood flow and to ensure neuronal activity and survival during the transatlantic non-stop flight. The birds must fast for 6 days during the long flight (Brown, 2014), and when glucose is in short supply, the brain increases ketone body metabolism. This may lead to a high demand for astrocytes to take up, synthesize and release fatty acids, which are alternative sources of energy that can be released as β-hydroxybutyrate, a ketone body that can fuel brain cells, including astrocytes, neurons, and oligodendrocytes (Achanta and Rae, 2017). Indeed, it has been demonstrated that higher levels βOHB in the blood, as is expected during the fasting period associated with the transatlantic flight, can meet all basal requirements and around half of all necessary energy for neuronal activity (Chowdhury et al., 2014). Notably, βOHB reduces the levels of reactive oxygen species associated with mitochondrial bioenergetic, we suggest that there may be an alternative pathway in Type II astrocytes using ketone bodies in association with the imposed fasting period of the long flight. However, because this alternative pathway does not support synaptic activity (Arakawa et al., 1991) we suggest that synaptic activity would depend on Type I astrocytes. Indeed, during neurotransmission, glutamate in the synaptic cleft activates its receptors on neurons and astrocytes before reuptake by astrocytes; this requires energy. Although the detailed glutamate uptake mechanism used by astrocytes remains controversial, astrocytes are known to store glycogen, particularly in areas with high synaptic activity, and glycogen-derived lactate can sustain neuronal activity, especially during hypoglycemia (Bolaños, 2016). Taken together, these findings support the idea that these distinct astrocyte metabolic pathways are differentially activated in Type I versus Type II astrocytes, inducing different levels of oxidative stress. Interestingly, more than 50% of Type III astrocytes (9 of 19) in wintering birds showed a complexity that was like that of Type I astrocytes in migrating birds and were unequivocally connected to blood vessels (**Table 1**). These cells seemed to have preserved their original morphology after the long-distance flight.

### Potential Limitations and Alternative Interpretations

However, it is not clear why hippocampal astrocytes morphology and numbers might differ between wintering and actively migrating birds.

Indeed, we must keep in mind that the glial morphologies of birds collected in August in the Bay of Fundy and September to March on Isla Canela are clearly different, but our discussion and interpretation so far, say that these differences are due to the long transatlantic flight, which occurred for these birds between August and September. Because the birds had spent at least 1 month (August to September) and perhaps as many as seven months (August to March) in Brazil, is the transatlantic flight the only possible cause of the differences that were found in glial morphology? To measure possible influence of capture dates on hippocampal astrocytes morphological complexities of wintering birds, we selected birds with closer capture dates and submit the sample of three or four animals captured in Bragança (Brazil) to the same statistical analysis. We found smaller changes in the mean values of hippocampal astrocytes morphological complexity which did not affect significantly the remarkable differences between hippocampal astrocytes morphologies of migrating and wintering birds detected with the whole sample (five birds). For some birds, however, the transatlantic flight was a long time in the past, and many other differences in the environment of Bay of Fundy and Isla Canela may change birds brain during this period. For example: (1) a different diet in Brazil versus the Bay of Fundy; (2) post-breeding physiological condition in the Bay of Fundy versus wintering or even pre-breeding physiological condition in Brazil; (3) a shortest flight from arctic Canada to the Bay of Fundy versus a very long flight from the Bay of Fundy to Brazil; and (4) very long days in the arctic summer versus 12-h days at the equator.

Thus, the evidence necessarily does not show the differences in glial cell numbers and morphologies are due to the transatlantic flight. Indeed, because cognitive activity, environmental enrichment, diet and metabolic stress are known to affect astrocytes morphophysiology, at least in mammals (Soffie et al., 1999; Diniz et al., 2010, 2012; Diniz D.G. et al., 2016; Rodríguez et al., 2014; Yeh et al., 2015; Tsai et al., 2016; Verkhratsky et al., 2016) it is difficult to distinguish their relative contributions to the morphological changes.

For example, there may be diet changes and glucocorticoids effects that may contribute to reduce potential environmental enrichment effects which may affect differentially, migrating and wintering bird’s metabolic pathways, with significant influences on astrocytes morphologies and number.

In addition, since the breeding range of semipalmated sandpipers is extensive, there is no reason to assume that the birds captured in Canada are from the same population as the birds captured in Brazil. Thus, another possibility to interpret our findings could be that the reported differences could also be related to potential breeding differences.

It is important to keep in mind that, based on morphological analysis of three selective astroglial markers (GFAP, glutamine synthetase, and S-100β), there is an emerging view that “astrocytes” comprise a heterogeneous population even within a given region (Rodríguez et al., 2014). Our findings limited to GFAP-immunolabeled astrocytes, may show only some of the morphological and numerical changes of astrocytes of the hippocampal formation of *C. pusilla* in response to a variety of environmental stimuli along the migratory route including that related to the intense exercise associated with the transatlantic flight.

Finally, it is important to recognize that because individual astrocytes were selected using systematic random sampling and the number of elements selected for reconstruction was rather large (499 in total; 250 in wintering birds and 249 in migrating birds), we expect no *a priori* sampling bias. Therefore, it is reasonable to propose that the proportions of reconstructed cells reflect the quantitative distribution of Type I and Type II astrocytes in the whole hippocampal formation of *C. pusilla*. Because we knew the total number of astrocytes from each animal (from stereological analysis) and also knew the proportion of Type I and Type II astrocytes expressed as percentage values of reconstructed cells in each group suggested by cluster analysis, we simply multiply the mean percentage values of each group by the total number of astrocytes to obtain the total numbers of Type I and Type II astrocytes in both wintering and migrating birds.

If this assumption is correct the total number of Type I and Type II astrocytes before (migrating birds) and after (wintering birds) the long-distance transatlantic flight are as follows: migrating sandpipers showed 118,621 Type II (76.7%) and 36,035 (23.3%) Type I astrocytes, whereas wintering birds showed 44,924 (63.6%) and 25,711 (36.4%) Type II and Type I astrocytes respectively. Taken together, these findings show that both Type I and Type II numbers were affected by the long journey but Type II astrocytes were more severely impacted.

Because we have no information in the literature about potential influence of sex and age on hippocampal astrocytes morphology in long-distance migratory birds, and we did not measure the age of individuals in our sample due to technical limitations, it is difficult to discuss these potential influences in detail. However, experience and sex are important variables that have been previously demonstrated to influence hippocampal-dependent tasks in birds (Astié et al., 2015; Rensel et al., 2015; Guigueno et al., 2016; Bingman and MacDougall-Shackleton, 2017), and migratory behavior is accompanied by hippocampal morphological changes including volume and neurogenesis (Barkan et al., 2016, 2017; de Morais Magalhães et al., 2017). Thus, these variables should be considered in future studies of hippocampal astrocytes morphologies in long-distance migratory birds.

Due to the correlational nature of the analysis, the differences in the morphologies and number of astrocytes, were associated to the role of the hippocampus as a center of multisensory integration using celestial and magnetic compasses for navigation. However, the story could be different if another higher order brain area, less involved in migration, exhibited similar astrocytes differences and this is a potential limitation of the present study, that could be avoided if another area was explored.

It is important to note that in previous work we detected an increase in volume in wintering *C. pusilla* when compared to migrating (de Morais Magalhães et al., 2017). In the present results this reduction in volume of hippocampal formation was not detected, suggesting that other cells of the hippocampal formation and associated neuropil may have effect over the volume and more data is necessary to clarify what generates the observed volume fluctuation.

### Environmental Changes and Astrocytes Morphology

The fall migration of the semipalmated sandpiper includes continental stopover sites and a multi-day non-stop flight across the Atlantic Ocean from northeastern North America to northeastern South America. The environment through which the birds fly, changes dramatically during this flight and birds probably integrate global cues, learned local gradient maps, and local landmark information in order to successfully complete migration (Bingman and Cheng, 2005; Mouritsen et al., 2016). Trans-oceanic and trans-continental long-distance navigation makes use of celestial and geomagnetic information (Frost and Mouritsen, 2006; Thorup and Holland, 2009) whereas learned gradient maps and local landmarks in various sensory modalities are associated with short distance overland migratory behavior (Biro et al., 2004; Frost and Mouritsen, 2006). Thus, migration may be considered as a kind of environmental enrichment which should contribute to increase astrocytes complexity (Ramírez-Rodríguez et al., 2014; Diniz D.G. et al., 2016). On the other hand, it has been demonstrated in mammals, that intense exercise, may cause excessive apoptosis and synapse plasticity damage through Ca^2+^ overload and endoplasmic reticulum stress-induced apoptosis pathway (Ding et al., 2014). These findings also demonstrated astrocytes activation after intense exercise which would explain astrocytes shrinkage we have found in wintering birds. Although these effects need to be confirmed in birds, it is a temptation to speculate that the intense exercise during the long-distance transatlantic non-stop flight could contribute to reduced morphological complexity and number of astrocytes. Because Type I astrocytes of migrating birds were affected in higher proportion than Type II, we suggested that in this subpopulation the beneficial effect of the environmental enrichment of migratory route may not compensate the negative influences induced by the intense exercise of the non-stop flight. In agreement our findings on *Charadrius semipalmatus* a shorebird which migrates over land with many stopovers, revealed an inverse correlation where wintering birds showed higher values of hippocampal astrocytes morphological complexities than that of migrating birds (unpublished results).

Thus, our findings from the hippocampus astrocytes of *C. pusilla* demonstrated a reduction in both morphological and number of GFAP immunolabeled astrocytes in wintering birds, suggesting that if the environmental enrichment along the journey does act to increase morphological complexity and number of astrocytes, its effects were not vigorous enough to be seen during the wintering period.

## Conclusion

Although our findings did not explain the genesis of the morphological and numerical astrocytes changes, many other studies have demonstrated that the form-function model is a good starting point for investigating the possible influences of multivariate factors on cell morphology and function. Because the evidence that Type I and Type II astrocytes may have distinct roles in the brain is indirect, and this study is explicitly correlational, the results simply raise important questions about the relationships between morphological and numerical changes in astrocytes and long-distance migration in shorebirds. The clear morphological and numerical differences we observed between migrating and wintering birds indicate that long-distance shorebird migrants may provide several opportunities to investigate a variety of questions about hippocampal astrocytes and migration.

## Author Contributions

All authors listed executed substantial contributions to the conception or design of the work; or the acquisition, analysis, or interpretation of data for the work; and drafting the work or revising it critically for important intellectual content; and final approval of the version to be published; agreed to be accountable for all aspects of the work in ensuring that questions related to the accuracy or integrity of any part of the work are appropriately investigated and resolved.

## Conflict of Interest Statement

The authors declare that the research was conducted in the absence of any commercial or financial relationships that could be construed as a potential conflict of interest.

## Supplementary Material

TABLE S1 Stereological parameters for the GFAP positive neurons in the left hippocampal formation of *Calidris pusilla*. ∑Q^-^ = equals the total number of objects of interest counted using the optical dissector, SSF = section sampling fraction, ASF = area sampling fraction, LHF = left hippocampal formation.

TABLE S2 Stereological parameters for the GFAP positive neurons in the right hippocampal formation of *C. pusilla*. ∑Q^-^ = equals the total number of objects of interest counted using the optical dissector, SSF = section sampling fraction, ASF = area sampling fraction, TSF = thickness sampling fraction, a(frame) = area of counting frame, A(x,y step) = grid size, RHF = right hippocampal formation.

TABLE S3 Stereological parameters for the GFAP positive neurons in the left hippocampal formation of *C. pusilla*. ∑Q^-^ = equals the total number of objects of interest counted using the optical dissector, SSF = section sampling fraction, ASF = area sampling fraction, TSF = thickness sampling fraction, a(frame) = area of counting frame, A(x,y step) = grid size, LHF = left hippocampal formation.

TABLE S4 Stereological parameters for the GFAP positive neurons in the right hippocampal formation of *C. pusilla*. ∑Q^-^ = equals the total number of objects of interest counted using the optical dissector, SSF = section sampling fraction, ASF = area sampling fraction, TSF = thickness sampling fraction, a(frame) = area of counting frame, A(x,y step) = grid size, RHF = right hippocampal formation.

DATA SHEET S2 Excel file shows all data related to astrocytes morphometric features with MMI > 0.55 and correspondent frequency distribution histograms for each morphometric variable. Because hierarchical cluster analysis was based on morphometric variables with multimodality index higher than 0.55, it is expected to find multimodal or at least bimodal distribution of morphometric features which were used to perform cluster analysis. Computationally, discriminant function analysis is very similar to multivariate analysis of variance (MANOVA). This procedure is used to determine whether groups differ with regard to the mean of a variable, and then to use that variable to predict group membership. The software used in the present report (Statistica 12) performed comparisons between a matrix of total variances and covariances, as well as between matrices of pooled within-group variances and covariances. These matrices were compared via multivariate *F*-tests in order to determine whether or not there are any significant differences (with regard to all variables) between groups. In addition, arithmetic mean and standard deviation were calculated for the variables chosen as best predictors for groups. Parametric statistical analysis using *t*-tests and non-parametric analysis using the Mann–Whitney *U*-test were applied for comparison between astrocytes morphological groups suggested by multivariate data analysis. Statistical significance was accepted at the 95% confidence level (*p* < 0.05). We further assessed our morphological classification using PERMANOVA and found similar results as described for cluster analysis.
