# Supplementary material for: Hippocampal Astrocytes in Migrating and Wintering Semipalmated Sandpiper Calidris pusilla
Source: Front Neuroanat. 2018 Jan 4;11:126. doi: 10.3389/fnana.2017.00126 (PMC5758497; doi:10.3389/fnana.2017.00126)
Supplement: TABLE S1 — Stereological parameters for the GFAP positive neurons in the left hippocampal formation of Calidris pusilla. ∑Q- = equals the total number of objects of interest counted using the optical dissector, SSF = section sampling fraction, ASF = area sampling fraction, LHF = left hippocampal formation. [file Data_Sheet_1.DOCX]

**S1 Table**: **Stereological parameters for the GFAP positive neurons in the left hippocampal formation of Calidris pusilla.** ΣQ- = Equals the total number of objects of interest counted using the optical dissector, SSF = Section Sampling Fraction, ASF = Area S, LHF= left hippocampal formation.

| **Animal** | **a(frame)**  **(µm)** | **A(x,y step)**  **(µm)** | **N° Counting**  **Frames** | **N°Sections LHF** | **ASF** | **SSF** | **TSF** | **ΣQ‐** |
| --- | --- | --- | --- | --- | --- | --- | --- | --- |
| ***C. pusilla* 82** | 110 x 80 | 120 x 120 | 691 | 10 | 0,61 | 0,17 | 0,72 | 3387 |
| ***C. pusilla* 100** | 110 x 80 | 120 x 120 | 994 | 09 | 0,61 | 0,17 | 0,75 | 4287 |
| ***C. pusilla* 102** | 110 x 80 | 120 x 120 | 1211 | 11 | 0,61 | 0,17 | 0,76 | 3783 |
| ***C. pusilla* 105** | 110 x 80 | 120 x 120 | 1262 | 18 | 0,61 | 0,17 | 0,76 | 5660 |
| ***C. pusilla* 89** | 110 x 80 | 120 x 120 | 1415 | 12 | 0,61 | 0,17 | 0,75 | 1844 |

**S2 Table: Stereological parameters for the GFAP positive neurons in the right hippocampal formation of *Calidris pusilla*.** ΣQ- = Equals the total number of objects of interest counted using the optical dissector, SSF = Section Sampling Fraction, ASF = Area Sampling Fraction, TSF = Thickness Sampling Fraction, a(frame) = Area of counting frame, A(x,y step) = grid size. RHF= Right hippocampal formation.

| **Animal** | **a(frame)**  **(µm)** | **A(x,y step)**  **(µm)** | **N° Counting**  **Frames** | **N°**  **Sections**  **RHF** | **ASF** | **SSF** | **TSF** | **ΣQ‐** |
| --- | --- | --- | --- | --- | --- | --- | --- | --- |
| ***C. pusilla* 82** | 110 x 80 | 120 x 120 | 994 | 13 | 0,61 | 0,17 | 0,72 | 4148 |
| ***C. pusilla* 100** | 110 x 80 | 120 x 120 | 948 | 10 | 0,61 | 0,17 | 0,74 | 4039 |
| ***C. pusilla* 102** | 110 x 80 | 180 x 180 | 463 | 10 | 0,27 | 0,17 | 0,74 | 1744 |
| ***C. pusilla* 105** | 110 x 80 | 120 x 120 | 1070 | 17 | 0,61 | 0,17 | 0,78 | 5023 |
| ***C. pusilla* 89** | 110 x 80 | 120 x 120 | 1360 | 11 | 0,61 | 0,17 | 0,78 | 2076 |

**S3 Table: Stereological parameters for the GFAP positive neurons in the left hippocampal formation of Calidris pusilla.** ΣQ- = Equals the total number of objects of interest counted using the optical dissector, SSF = Section Sampling Fraction, ASF = Area Sampling Fraction, TSF = Thickness Sampling Fraction, a(frame) = Area of counting frame, A(x,y step) = grid size LHF= left hippocampal formation.

| **Animal** | **a(frame)**  **(µm)** | **A(x,y step)**  **(µm)** | **N° Counting**  **Frames** | **N° Sections LHF** | **ASF** | **SSF** | **TSF** | **ΣQ‐** |
| --- | --- | --- | --- | --- | --- | --- | --- | --- |
| ***C. pusilla* 10** | 110 x 80 | 200 x 200 | 607 | 18 | 0,22 | 0,17 | 0,75 | 3.607,00 |
| ***C. pusilla* 12** | 110 x 80 | 200 x 200 | 704 | 17 | 0,22 | 0,17 | 0,73 | 4.995,00 |
| ***C. pusilla* 14** | 110 x 80 | 200 x 200 | 574 | 19 | 0,22 | 0,17 | 0,72 | 5.184,00 |
| ***C. pusilla* 16** | 110 x 80 | 200 x 200 | 435 | 16 | 0,22 | 0,17 | 0,71 | 1.937,00 |
| ***C. pusilla* 21** | 110 x 80 | 200 x 200 | 565 | 13 | 0,22 | 0,17 | 0,70 | 2.622,00 |

**S4 Table: Stereological parameters for the GFAP positive neurons in the right hippocampal formation of *Calidris pusilla*.** ΣQ- = Equals the total number of objects of interest counted using the optical dissector, SSF = Section Sampling Fraction, ASF = Area Sampling Fraction, TSF = Thickness Sampling Fraction, a(frame) = Area of counting frame, A(x,y step) = grid size. RHF= Right hippocampal formation.

| **Animal** | **a(frame)**  **(µm)** | **A(x,y step)**  **(µm)** | **N° Counting**  **Frames** | **N° Sections RHF** | **ASF** | **SSF** | **TSF** | **ΣQ‐** |
| --- | --- | --- | --- | --- | --- | --- | --- | --- |
| ***C. pusilla* 10** | 110 x 80 | 200 x 200 | 562 | 18 | 0,22 | 0,17 | 0,75 | 3.679,00 |
| ***C. pusilla* 12** | 110 x 80 | 200 x 200 | 772 | 17 | 0,22 | 0,17 | 0,71 | 5.234,00 |
| ***C. pusilla* 14** | 110 x 80 | 200 x 200 | 686 | 20 | 0,22 | 0,17 | 0,74 | 3.972,00 |
| ***C. pusilla* 16** | 110 x 80 | 200 x 200 | 487 | 16 | 0,22 | 0,17 | 0,67 | 3.644,00 |
| ***C. pusilla* 21** | 110 x 80 | 200 x 200 | 677 | 13 | 0,22 | 0,17 | 0,68 | 3.048,00 |
